# A Novel Early-Stage Lung Adenocarcinoma Prognostic Model Based on Feature Selection With Orthogonal Regression

**DOI:** 10.3389/fcell.2020.620746

**Published:** 2021-01-08

**Authors:** Binhua Tang, Yuqi Wang, Yu Chen, Ming Li, Yongfeng Tao

**Affiliations:** Epigenetics & Function Group, Hohai University, Nanjing, China

**Keywords:** early stage, prognosis, feature selection, orthogonal regression, LASSO

## Abstract

Carcinoma diagnosis and prognosis are still hindered by the lack of effective prediction model and integration methodology. We proposed a novel feature selection with orthogonal regression (FSOR) method to resolve predictor selection and performance optimization. Functional enrichment and clinical outcome analyses with multi-omics information validated the method's robustness in the early-stage prognosis of lung adenocarcinoma. Furthermore, compared with the classic least absolute shrinkage and selection operator (LASSO) regression method [the averaged 1- to 4-years predictive area under the receiver operating characteristic curve (AUC) measure, 0.6998], the proposed one outperforms more accurately by 0.7208 with fewer predictors, particularly its averaged 1- to 3-years AUC reaches 0.723, vs. classic 0.6917 on The Cancer Genome Atlas (TCGA). In sum, the proposed method can deliver better prediction performance for early-stage prognosis and improve therapy strategy but with less predictor consideration and computation burden. The self-composed running scripts, together with the processed results, are available at https://github.com/gladex/PM-FSOR.

## Introduction

Lung adenocarcinoma (LUAD), an important subtype in lung carcinoma (Chen et al., 2020), is one of the most malignant and widely spread cancers in the world (Jemal et al., 2011), with its incidence increased considerably in recent years (Xu et al., 2020). SEER Cancer Statistics Review suggests that the survival rate of LUAD is extremely low; specifically, the 1-year survival rate is lower than 50%, and the 5-years survival rate is around 18% (Siegel et al., 2020).

In the last decades, high-throughput sequencing technologies in cancer genomics and epigenomics have created ever greatest possibilities to improve clinical diagnosis and prognosis (Gao et al., 2018; Li S. et al., 2018). Recently, Li T. et al. (2018) constructed a protein–protein interaction (PPI) network with differentially expressed genes (DEGs) to determine hub genes. Wang et al. (2020) combined independent-sample and paired-sample experiments to determine prognostic markers in LUAD. Guo et al. (2019) integrated PPI network and enrichment analysis to screen functional DEGs.

However, due to the relatively small sample size and different profiling platforms utilized, the analysis results in those studies may not be invariably consistent. The meta-analysis has been demonstrated as a feasible approach to integrate and explore such multi-source information. Silva et al. (2019) performed a meta-analysis of transcriptomics to investigate Schwann cell reprogramming and lung cancer progression. Selvaraj et al. (2018) conducted meta-analysis on three LUAD gene profiling datasets and identified target genes related to poor overall prognosis.

Besides, survival prediction from high-dimensional gene profiling and clinical information poses challenges in cancer studies (Tang et al., 2019), the key of which is the collinearity in high-dimensional profiling data. Thus, several feature selection methods were developed and utilized so far. Tibshirani (1997) proposed a penalized least absolute shrinkage and selection operator (LASSO) regression method in a Cox model, which is already a classic method of constructing survival models for high-dimensional data in the past decades. Simon et al. (2013) proposed a sparse group LASSO. Mittal et al. (2013) applied regularized parametric regression in survival analysis. In most methods, least-square regression was adopted to classify the correlation between feature and prediction. Recently Zhang et al. (2018) retained more statistical and structural information by restricting least-square regression into orthogonal regression. Then Wu et al. proposed a feature selection method with orthogonal regression and applied it to the image feature extraction.

Here, we carried out a meta-analysis to identify potential biomarkers of survival-related genes in LUAD. Based on the four LUAD gene profiling datasets retrieved from Gene Expression Omnibus (GEO), a total of 1,208 up-regulated DEGs were identified. The method based on feature selection with orthogonal regression (FSOR) was proposed to rank all feature genes with a weighted matrix. PPI network analysis was conducted to further screen genes with molecular function (MF) and mechanism. The gene profiling and clinical information from The Cancer Genome Atlas (TCGA) were finally retrieved to construct a prognostic model with a stepwise multivariate Cox regression method. A total of eight hub genes specific to the poor LUAD prognosis were identified in this study. Together, a performance comparison between the proposed FSOR and classic LASSO methods was deployed in feature selection.

## Materials and Methods

### A Novel Approach Proposed for Meta-Analysis and Model Construction

To systematically determine prognostic signatures in LUAD, we firstly conducted a meta-analysis based on gene expression profiling data to identify candidate DEGs before further integrating with clinical information.

**Figure 1:**
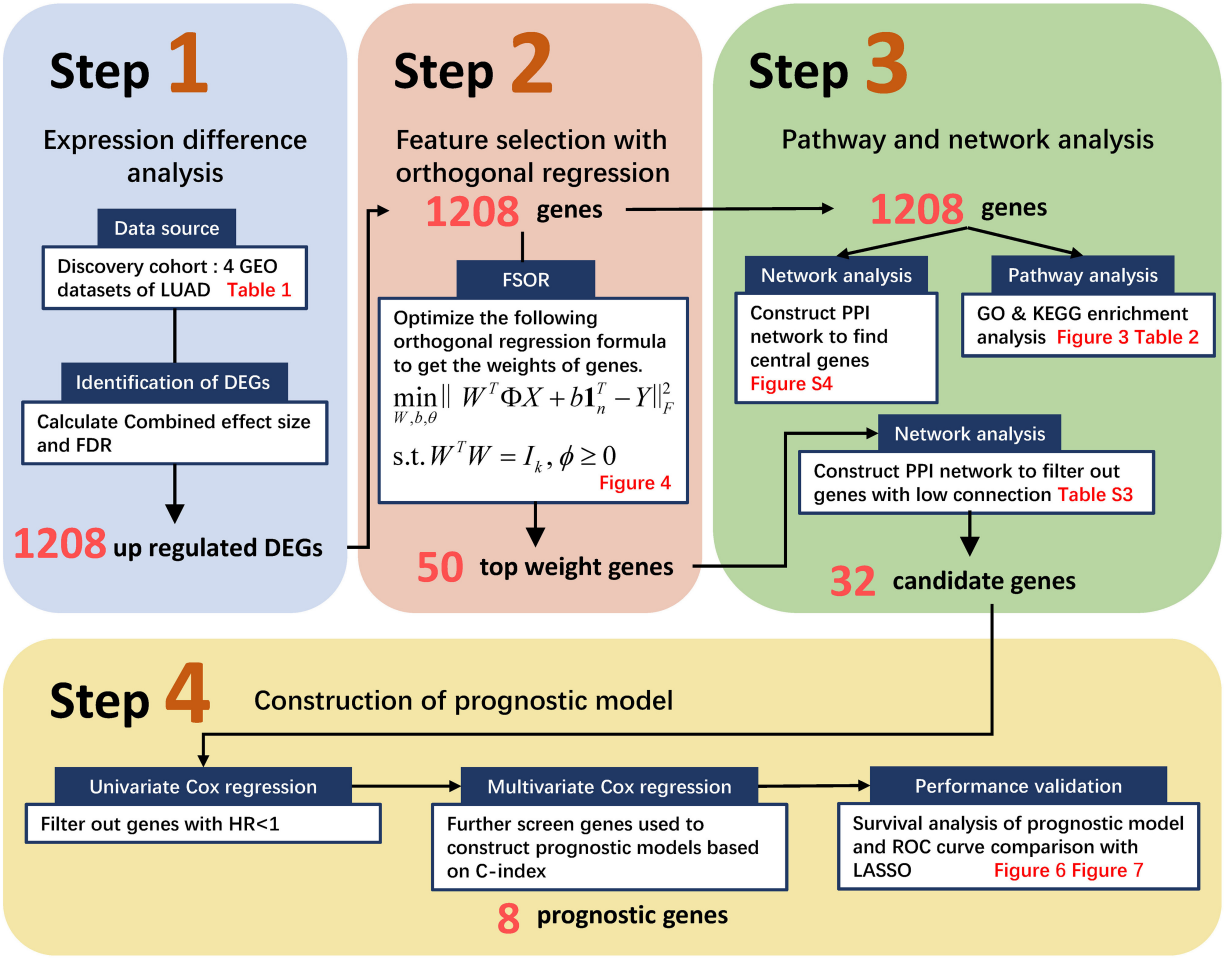
The flowchart to screen candidate genes and construct a prognostic model based on the proposed feature selection with orthogonal regression (FSOR) approach. It ranges from retrieving Gene Expression Omnibus (GEO) profiling information, gene filtering, proposing the FSOR method in predictor selection, and univariate and multivariate Cox regression to performance validation via survival prediction and receiver operating characteristic (ROC) measures.

Generally, the pipeline contains four major procedures as illustrated in Figure 1, ranging from pre-processing raw GEO data to constructing an orthogonal regression-based prognosis model and its performance validation. Namely, Step 1 retrieved the four GEO expression profiling datasets and filtered out candidate DEGs with the preset Combined Effective Size (CombinedES) and false discovery rate (FDR); Step 2 proposed the method to filter candidate gene predictors based on FSOR; in Step 3, functional pathway and network analysis eventually identified 32 candidate predictors for the prognosis model. Step 4 involved univariate and multivariate Cox regression, and further performance comparison with classic LASSO was also implemented based on predicted survival rate and its corresponding area under the receiver operating characteristic (ROC) curve (AUC) measure. The statistical comparison validated the effectiveness of the proposed method.

We used NetworkAnalyst (Zhou et al., 2019) to find DEGs through combined effect size model. To further screen these genes, we utilized and PPI network to examine the DEGs on the underlying association among gene expression, clinical information, and molecular mechanism.

A total of 32 up-regulated DEGs were screened as candidate genes to construct the prognostic model. With gene expression and clinical data from TCGA (Weinstein et al., 2013), we further performed a multivariate Cox regression on the candidate genes, and we finally confirm eight gene predictors to construct the prognostic model, together with the corresponding risk score information.

### Data Source

The gene expression profiles for LUAD were downloaded from GEO. We searched the profiling data using the combined strategy, such as “LUAD” and “lung adenocarcinoma” [key words], “homo sapiens” [organism], and “expression profiling by array” [study type]. A total of four LUAD expression profiles (GSE32036, GSE32867, GSE33532, and GSE75037) were retrieved from GEO. And the corresponding accession number, platform, and sample information are listed in Table 1.

**Table 1:** Summary information of the GEO datasets in the analysis.

**Dataset ID**	**Profiling platform information**	N^*^	T^*^
GSE32036	GPL6844; Illumina HumanWG-6 v3.0 expression bead chip	59	73
GSE32867	GPL6844; Illumina HumanWG-6 v3.0 expression bead chip	58	58
GSE33532	GPL570; Affymetrix Human Genome U133 Plus 2.0 Array	20	40
GSE75037	GPL6844; Illumina HumanWG-6 v3.0 expression bead chip	83	83

* *Numbers of Normal and Tumor (LUAD) samples retrieved from GEO*.

### Adjustment of Batch Effect and Identification of Differentially Expressed Genes

Raw GEO data retrieved were preprocessed with grouping samples and annotating probe IDs, according to the clinical information and platform information. The web-tool NetworkAnalyst was utilized to remove the batch effect and identify the DEGs between normal and tumor samples. Expression level in each dataset was normalized by the log2 transformation.

**Figure 2:**
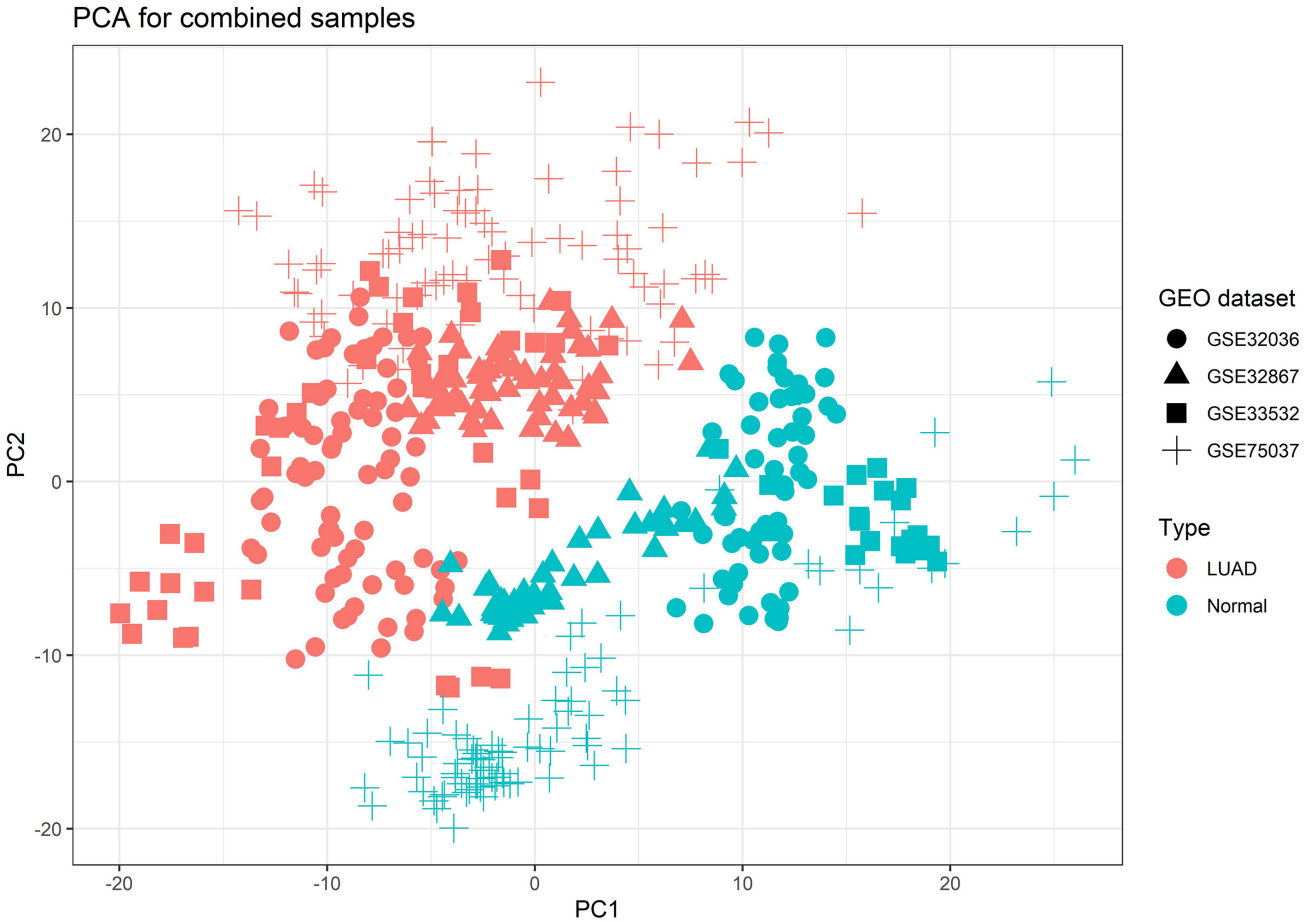
Principal component analysis (PCA) result for the combined samples of the four lung adenocarcinoma (LUAD) studies from Gene Expression Omnibus (GEO), which explicitly brought out emphasized variation and separation pattern between tumor and normal samples.

Due to the raw datasets from different profiling platforms, the underlying batch effect was initially removed, and then the datasets were calculated for the combined effect sizes (CombinedES). Effect size represents the difference between group means divided by standard deviation, considered as combinable and comparable across different studies. We chose a random effects model (REM) to calculate the CombinedES of each annotated gene. In REM, each study contains a random effect that can incorporate alien cross-study heterogeneity caused by diverse platforms. With the FDR set at 0.05 and the cutoff at |CombinedES| > 1.0, 2,320 DEGs were filtered, specifically, 1,208 up-regulated DEGs with CombinedES > 1.0 and 1,112 down-regulated DEGs with CombinedES < −1.0. Figure 2 depicts the principal component analysis (PCA) on the combined samples from the four LUAD studies.

### Supervised Feature Selection With Orthogonal Regression

To determine survival predictors from the up-regulated DEGs, we proposed a novel orthogonal regression method for feature extraction, different from classic least-square-based linear regression approaches.

To measure the feature's importance level, a weighted projection matrix was introduced to the orthogonal regression method. Thus, features can be ranked according to the respective weights by minimizing the below regression equation:

(1) $$\begin{array}{l} \underset{W,b,\theta }{\min}||W^{T}\Phi X+b1_{n}^{T}-Y{||}_{F}^{2}\\ \text{s}.\text{t}W^{T}W=I_{k},\Phi \geq 0 \end{array}$$

where *W* ∈ *R*^*d*×*k*^ denotes an orthogonal projection matrix, namely, $W^{T}W=I_{k}$, Φ ∈ *R*^*d*×*d*^ a weighted diagonal matrix with ϕ in the diagonal, *X* ∈ *R*^*d*×*n*^ the input matrix, *b* ∈ *R*^*k*×1^ the bias vector, **1**_*n*_=[1,1,...,1]^*T*^ ∈ *R*^*n×1*^, *Y* ∈ *R*^*k*×*n*^ the label matrix, and ||·||_*F*_ the Frobenius norm of a matrix, defined as $||A{||}_{F}=\sqrt{\sum_{i=1}^{m}\sum_{j=1}^{n}|{a_{i}}_{j}{|}^{2}}$; and *d, n*, and *k* represent the counts of features, samples, and labels categories, respectively.

In this study, the log2-transformed expression data for a total of 1,060 up-regulated genes across 479 samples were utilized as the input matrix *X*; and one-hot encoding of clinical survival status, together with survival time information, was combined into the label matrix *Y*. For the limit solution, the partial derivative of Equation (1) concerning *b* is as follows:

(2) $$\frac{\partial {\left\|W^{T}\Phi X+b1_{n}^{T}-Y\right\|}_{F}^{2}}{\partial b}=0$$

and then $b=\frac{1}{n}\left(Y1_{n}-W^{T}\Phi X1_{n}\right)$, and Equation (1) can be reformatted as

(3) $$\underset{W,\theta }{\min}\;{\left\|W^{T}\Phi XM-YM\right\|}_{F}^{2},\text{s.t. }W^{T}W=I_{k},\;\phi \geq 0$$

where $M=I_{n}-\left(1/n\right)1_{n}1_{n}^{T}$. Thus, we approximate this optimization problem by tuning two parameters separately. When Φ is fixed, we can update *W* by the following:

(4) $$\begin{array}{l} \underset{W^{T}W=I_{k}}{\min}Tr\left(W^{T}CW-2W^{T}D\right) \end{array}$$

where *C* = Φ*XMX*^*T*^Φ^*T*^ and *D* = Φ*XMY*^*T*^.

Equation (4) is referred to a quadratic problem on the Stiefel manifold. Nie et al. (2017) proposed a novel generalized power iteration (GPI) method for solving this problem (see Supplementary Material); thus, it can be reformatted as

(5) $$\begin{array}{l} \underset{W^{T}W=I_{k}}{\max}\text{Tr}\left(W^{T}\tilde{C}W\right)+2\text{Tr(}{\text{W}}^{T}\text{D)} \end{array}$$

where $\tilde{C}=\alpha I_{d}-C$, and α is a relaxation parameter to guarantee $\tilde{C}$ positive definite. We set α as the dominant eigenvalue of *C*.

When *W* fixed, Φ is updated as below:

(6) $$\begin{array}{l} \underset{W,b,\theta }{\min}\left[Tr\left(\Phi XMX^{T}\Phi WW^{T}\right)-Tr\left(2\Phi XMY^{T}W^{T}\right)\right]\\ \text{s}.\text{t}.\;W^{T}W=I_{k},\;\phi \geq 0 \end{array}$$

Following the diagonal matrix lemma, there exists Tr(*ABAC*) = *a*^*T*^(*B*^*T*^ ° *C*)*a* for a diagonal *A*, where α denotes the leading diagonal vector in *A*, *B*^*T*^ ° *C* the Hadamard product of two matrixes. Thus, Equation (6) can be reformed as

(7) $$\begin{array}{l} \underset{\phi \geq 0}{\min}\phi^{T}H\phi -\phi^{T}r \end{array}$$

where *H* = (*XM*^*T*^*X*^*T*^) ° (*WW*^*T*^) and *r* = diag(2*XMY*^*T*^*W*^*T*^).

The constrained minimization problem can be solved by an augmented Lagrangian multiplier (ALM) method (Hu et al., 2019); see Supplementary Material. The ALM function for Equation (7) is

(8) $$\begin{array}{l} L(\phi ,v,\mu ,\lambda_{1},\lambda_{2})=\phi^{T}H\phi -\phi^{T}r+\frac{\mu }{2}{\left\|\phi -v+\frac{1}{\mu }\lambda_{1}\right\|}_{F}^{2}\\ +\frac{\mu }{2}(\phi^{T}1_{d}-1+\frac{1}{\mu }\lambda_{2}{)}^{2}\\ \text{s.t. }v\geq 0 \end{array}$$

where ν and λ_1_ are column vectors, and μ and λ_2_ are variables of the Lagrangian function.

When ν fixed, Equation (7) is converted as

(9) $$\begin{array}{l} \underset{\phi }{\min}\frac{1}{2}\phi^{T}J\phi -\phi^{T}g \end{array}$$

where and *g* = μ*v* + μ1_*d*_ − λ_2_**1**_*d*_ − λ_1_ + *r*. And ϕ is estimated as $\hat{\phi }=J^{-1}g$.

When ϕ fixed, Equation (7) can be reformulated as

(10) $$\begin{array}{l} \underset{v\geq 0}{\min}{\left\|v-(\phi +\frac{1}{\mu }{\text{λ}}_{1})\right\|}^{2} \end{array}$$

Then, ν is estimated as

(11) $$\hat{v}=f(\hat{\phi },\mu ,\lambda_{1})=\{\begin{array}{l} \hat{\phi }+\frac{1}{\mu }\lambda_{1},\;\hat{\phi }+\frac{1}{\mu }\lambda_{1}\geq 0\\ \;0\;,\;\hat{\phi }+\frac{1}{\mu }\lambda_{1}<0 \end{array}$$

By combining the two methods, we can get the optimal solution for the orthogonal projection matrix *W* ∈ *R*^*d*×*k*^ and the weighted diagonal matrix Φ ∈ *R*^*d*×*d*^. The pseudocode for solving the optimization problem in Equation (4) is depicted as below,


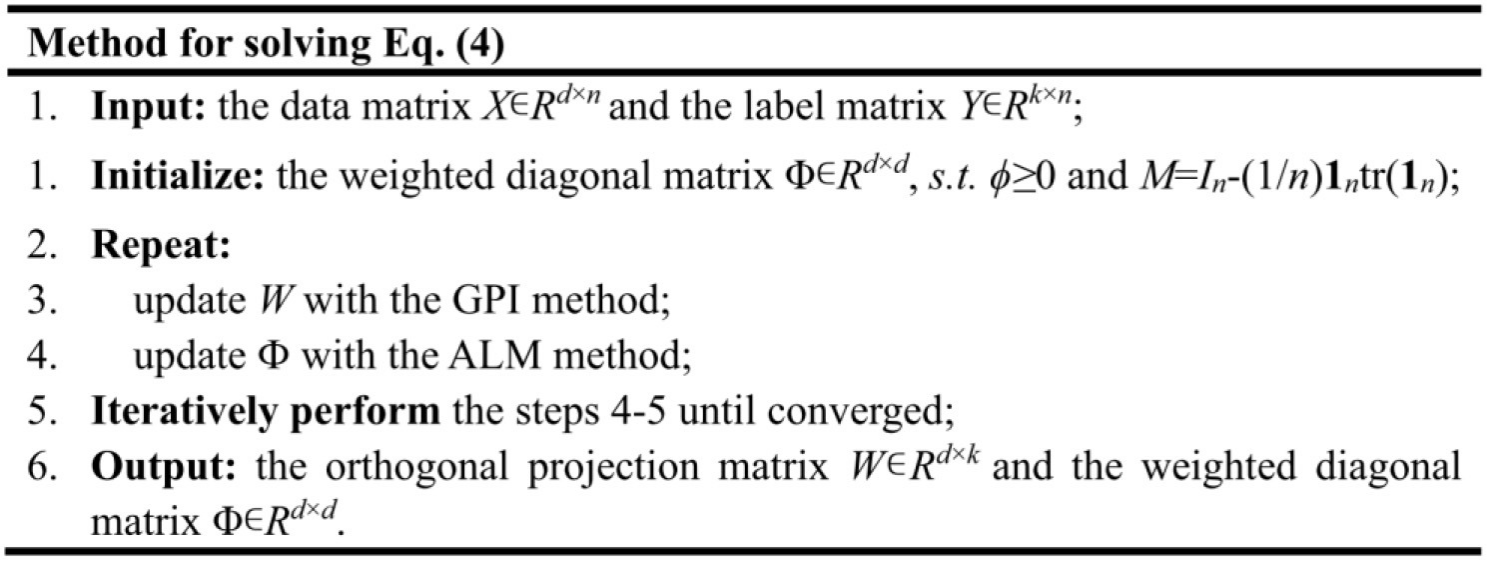


The features with higher weights will be filtered out through sorting the obtained ϕ. Thus, the number of screened features can be further customized to facilitate subsequent analysis.

### Functional Evaluation via Protein–Protein Interaction Network

To evaluate the underlying biological function, PPIs among the FSOR-filtered genes were further predicted with STRING (Szklarczyk et al., 2018). We chose gene nodes with at least one edge connected in the PPI network as candidates for further analysis. Connectivity within the PPI network nodes is adopted to evaluate the underlying function and further screen for prognostic genes (Li S. et al., 2018). In this study, cytoHubba in Cytoscape is utilized to detect nodes with a high degree, namely, central nodes in a PPI network, where maximal clique centrality (MCC) is adopted to rank the identified central nodes.

### Multivariate Cox Regression and Prognostic Model Construction

We adopted the survival package to construct a multivariate Cox regression model. The covariate count in the model was optimized by stepwise regression, and specifically, the Bayesian information criterion (BIC) was chosen to refine the covariate combination.

A total of eight candidate genes were finally screened as predictors to construct the prognostic model, where risks of a specific endpoint from the predictors can be calculated for an individual patient.

Furthermore, to explore the relationship between candidate gene predictors and clinical survival information in LUAD, a risk score function *h*(*t*) is introduced with multiple covariates from the Cox regression prognostic model, depicted as

(12) $$\begin{array}{r} h\left(t\right)=h_{0}\left(t\right)\times \exp(\beta_{1}\times {\text{expr}}_{1}+\beta_{2}\times {\text{expr}}_{2}+\cdots \\ +\beta_{m}\times {\text{expr}}_{m}) \end{array}$$

where *h*(*t*) denotes a risk score of mortality at time *t, h*_0_(*t*) the base value of risk score, β_*m*_ the coefficient of gene *m*, and expr_*m*_ the expression data of gene *m*. Based on risk score, samples are to segmented into two or more groups, namely, the high-risk group and low-risk group, etc. The risk score of the derived prognostic model can be depicted as;

(13) $$\begin{array}{l} \text{Risk score}=\left(1.1168\times \text{exp}{\text{r}}_{\text{RACGAP1}}\right)\\ +\left(0.4740\times \text{exp}{\text{r}}_{\text{CDCA8}}\right)\\ +\left(1.4432\times \text{exp}{\text{r}}_{\text{RCC2}}\right)+\left(1.7456\times \text{exp}{\text{r}}_{\text{PLK1}}\right)\\ +\left(1.6966\times \text{exp}{\text{r}}_{\text{KIF20B}}\right)\\ +\left(1.1153\times \text{exp}{\text{r}}_{\text{ALG3}}\right)+\left(0.6784\times \text{exp}{\text{r}}_{\text{BRCA1}}\right)\\ +\left(1.3090\times \text{exp}{\text{r}}_{\text{CHAF1B}}\right) \end{array}$$

## Results

### Gene Ontology and Kyoto Encyclopedia of Genes and Genomes Pathway Analysis of Differentially Expressed Genes

**Figure 3:**
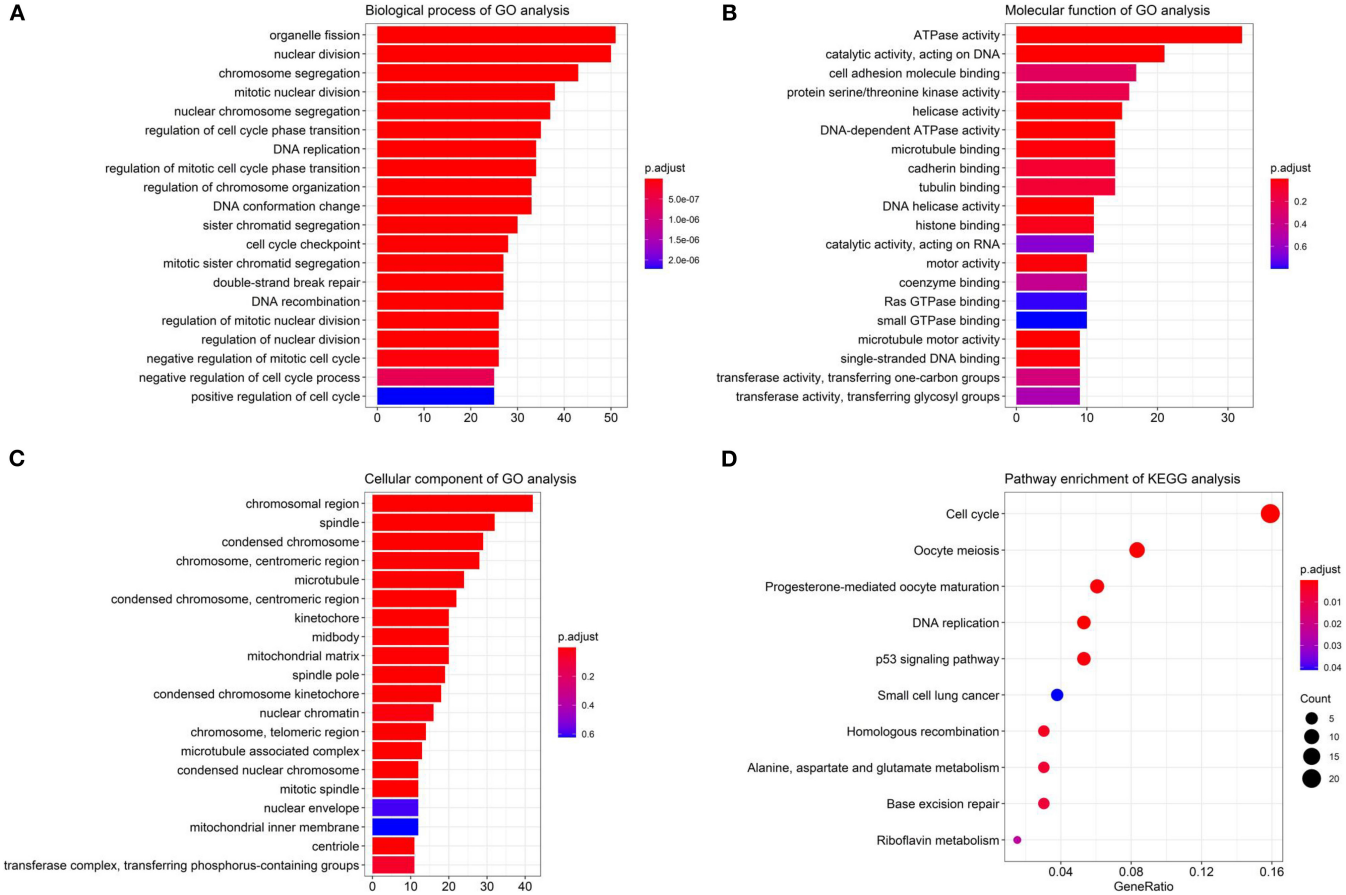
The Gene Ontology (GO) and Kyoto Encyclopedia of Genes and Genomes (KEGG) enrichment analyses. **(A)** The biological process (BP); **(B)** the molecular function (MF); **(C)** the cellular component (CC) terms; **(D)** the enrichment of the KEGG pathways.

We performed the Gene Ontology (GO) function and Kyoto Encyclopedia of Genes and Genomes (KEGG) pathway enrichment analyses on the identified DEGs, including biological process (BP), MF, and cellular component (CC). The BP analysis showed that the DEGs were enriched in chromosome segregation, nuclear division, organelle fission, and sister chromatid segregation. The CC analysis showed that the DEGs were enriched in chromosomal region, chromosome, centromeric region, condensed chromosome, and kinetochore. The MF analysis showed that the DEGs were enriched in catalytic activity, ATPase activity, and chromatin binding, as listed in Figure 3.

Furthermore, in Figure 3D, the KEGG pathway analysis showed that DEGs were enriched in such procedures as cell cycle, DNA replication, oocyte meiosis, and progesterone-mediated oocyte maturation, closely related to carcinoma development.

Table 2 lists the detailed information for the top four items in each category, filtered from the GO and KEGG enrichment analyses results for the DEGs in LUAD.

**Table 2:** Result of GO and KEGG enrichment analyses.

**Category**	**Term**	**Description**	**Count**	***P*-value**
BP term	GO:0007059	Chromosome segregation	39	1.77E-23
BP term	GO:0000280	Nuclear division	43	6.33E-23
BP term	GO:0048285	Organelle fission	44	4.59E-22
BP term	GO:0000819	Sister chromatid segregation	29	5.50E-21
CC term	GO:0098687	Chromosomal region	41	8.77E-22
CC term	GO:0000793	Condensed chromosome	27	6.79E-16
CC term	GO:0000775	Chromosome, centromeric region	26	9.96E-16
CC term	GO:0000776	Kinetochore	20	4.46E-13
MF term	GO:0140097	Catalytic activity, acting on DNA	13	9.59E-06
MF term	GO:0008094	DNA-dependent ATPase activity	9	1.11E-05
MF term	GO:0005509	ATPase activity	16	1.39E-04
MF term	GO:0003682	chromatin binding	21	1.57E-04
KEGG Pathway	hsa04110	Cell cycle	21	2.36E-13
KEGG Pathway	hsa03030	DNA replication	7	1.01E-05
KEGG Pathway	hsa04114	Oocyte meiosis	11	3.42E-05
KEGG Pathway	hsa04914	Progesterone-mediated oocyte maturation	8	5.81E-04

### Identifying the Candidate Genes With Both Feature Selection With Orthogonal Regression and Protein–Protein Interaction Approaches

We performed the FSOR analysis to weight features on all 1,208 up-regulated genes across 479 LUAD samples from TCGA, with the convergence condition between two FSOR consecutive iterations set ≤ 0.1.

**Figure 4:**
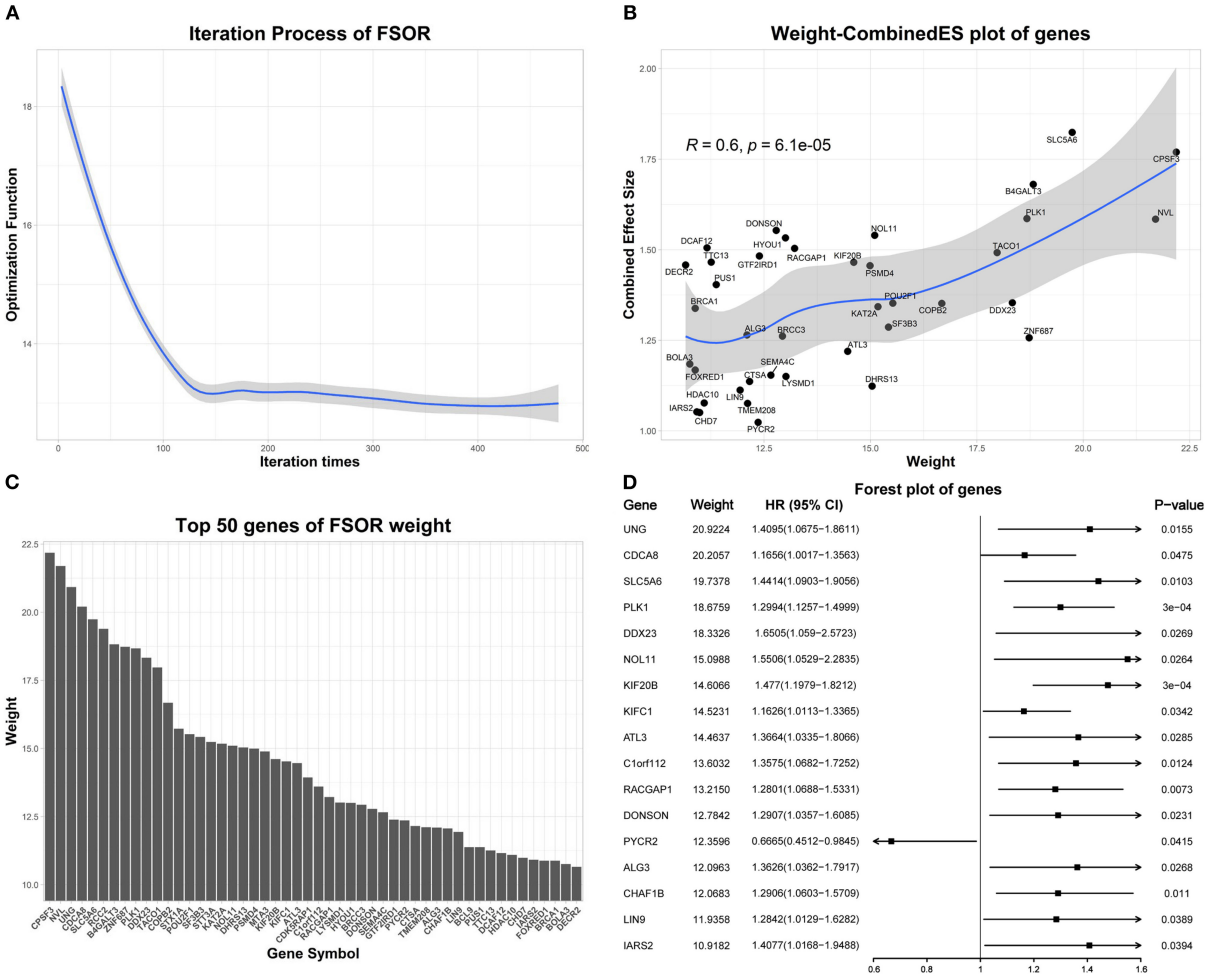
The feature selection with orthogonal regression (FSOR) analysis results on the differentially expressed genes (DEGs). **(A)** The optimization procedure convergences to 13.01 after 477 iterations. **(B)** Top 50 genes manifesting the statistical concordance between their derived FSOR weights and corresponding Combined Effective Size (CombinedES) values. **(C)** The derived weight distribution for the top 50 genes. **(D)** The initial univariate Cox regression on the top 50 DEGs revealed 17 among them that were statistically significant (*p*-value ≤ 0.05).

Figure 4 depicts the detailed FSOR analysis results. The Pearson correlation test was performed on the weight and combined effect size of genes in Figure 4B. LOESS regression was used to fit these points, and the shaded area represents a 95% confidence interval of the regression fitting curve. Figure 4D represents the univariate Cox regression results of top-weighted genes.

Based on the FSOR analysis, the top 50 genes were chosen from its output weighted matrix. To ensure the underlying functional association among the selected genes, PPIs were further predicted with STRING (Szklarczyk et al., 2018), listed in Supplementary Material. From the derived PPI network, we chose the gene nodes with at least one edge connected within the network; thus, a total of 32 genes were identified as candidate genes; see details in Supplementary Material.

### Univariate and Multivariate Cox Regression of the Candidate Genes

The validation data for the Cox regression were retrieved from TCGA. In Cox regression modeling, the coxph function in the survival package was adopted to calculate the bias coefficient (β), hazard ratio (HR), and *p*-value.

The genes with HR ≥ 1 in univariate Cox regression were filtered out for further multivariate Cox regression. With the step function, we optimize the number of covariates based on c-index in the Cox regression model. Thus, eight genes were determined as the candidate predictors in formulating the prognostic model, as depicted in Table 3.

**Table 3:** Summary of the univariate and multivariate Cox regression.

**Gene symbol**	**Univariate analysis**		**Multivariate analysis**	
	**HR (95% CI)**	***P*-value**	**HR (95% CI)**	***P*-value**
CPSF3	1.1262 (0.7638–1.6605)	0.5485		
UNG	1.4095 (1.0675–1.8611)	0.0155		
CDCA8	1.1656 (1.0017–1.3563)	0.0475	0.474 (0.305–0.737)	0.0009
RCC2	1.2477 (0.9487–1.6408)	0.1133	1.443 (0.973–2.141)	0.0684
PLK1	1.2994 (1.1257–1.4999)	0.0003	1.746 (1.230–2.476)	0.0018
DDX23	1.6505 (1.059–2.5723)	0.0269		
TACO1	1.1527 (0.7785–1.7069)	0.4779		
COPB2	1.3559 (0.9071–2.0268)	0.1376		
STX1A	0.9996 (0.8782–1.1379)	0.9953		
POU2F1	1.0808 (0.8968–1.3026)	0.4143		
SF3B3	1.2231 (0.8769–1.7061)	0.2356		
STT3A	1.1578 (0.8355–1.6043)	0.3787		
KAT2A	0.8785 (0.6924–1.1146)	0.2861		
PSMD4	1.1397 (0.7998–1.6241)	0.4692		
MTA3	0.9545 (0.7245–1.2576)	0.7408		
KIF20B	1.477 (1.1979–1.8212)	0.0003	1.697 (1.201–2.397)	0.0027
KIFC1	1.1626 (1.0113–1.3365)	0.0342		
C1orf112	1.3575 (1.0682–1.7252)	0.0124		
RACGAP1	1.2801 (1.0688–1.5331)	0.0073	1.117 (0.747–1.670)	0.5903
BRCC3	1.0651 (0.7323–1.549)	0.7416		
DONSON	1.2907 (1.0357–1.6085)	0.0231		
GTF2IRD1	0.8124 (0.6071–1.087)	0.1619		
ALG3	1.3626 (1.0362–1.7917)	0.0268	1.115 (0.817–1.523)	0.4919
CHAF1B	1.2906 (1.0603–1.5709)	0.011	1.309 (0.940–1.824)	0.1114
LIN9	1.2842 (1.0129–1.6282)	0.0389		
BCL9	1.0935 (0.8659–1.3809)	0.4527		
PUS1	1.0632 (0.8169–1.3837)	0.6485		
HDAC10	0.8455 (0.65–1.0998)	0.2111		
CHD7	1.106 (0.9273–1.3191)	0.2623		
FOXRED1	0.8799 (0.6521–1.1874)	0.4029		
BRCA1	1.1587 (0.9857–1.3622)	0.0742	0.678 (0.483–0.952)	0.0249
BOLA3	1.0905 (0.8617–1.3802)	0.4708		

### Cross-Validation by the Protein–Protein Interaction Network and Clique Centrality Analyses

To determine whether there exist the protein-level functional associations among the candidate gene predictors, the PPI network analysis was conducted on 32 candidate genes subsequently. Among them, the eight genes were selected for constructing the prognostic model after univariate and multivariate regression analyses.

**Figure 5:**
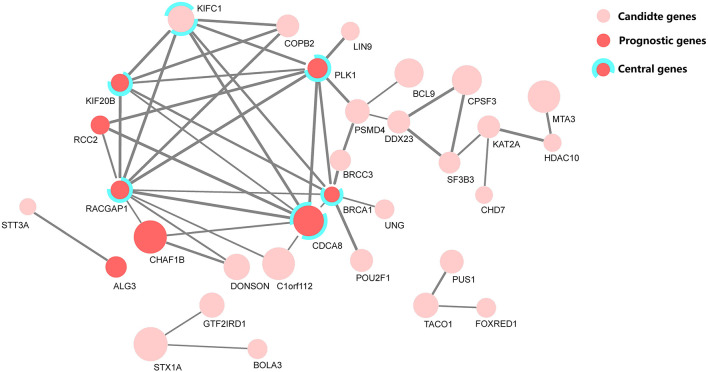
The protein–protein interaction (PPI) network of 32 candidate genes, where eight genes were selected as predictors in the prognostic model. Among them, five genes were defined as central genes by maximal clique centrality (MCC). The node size denotes the fold-change level of each differentially expressed gene, and the edge width denotes the combined score, a statistical confidence level calculated with STRING (Szklarczyk et al., 2018).

Research on prognosis-related genes in recent years usually took the gene connectivity in a PPI network into consideration (Guo and Li, 2019; Li et al., 2020). Here, all up-regulated genes were firstly calculated for their MCC values (Chin et al., 2014), and then the top 100 genes based on the sorted MCC values were defined as central nodes to cross-validate the functional association among these prognostic genes, together with the PPI network. Figure 5 depicts the identified PPI network of 32 candidate genes, marked with central and prognostic genes.

From the cross-validation diagram in Figure 5, it is evident that the identified predictors have a significant concordance in their biological function and network clique centrality property; namely, five out of eight predictors are both prognostic and central genes.

### Validation of the Prognostic Model With Clinical Survival Analyses

To determine and validate the statistical association between the risk model predictor and clinical outcome, survival analyses on the identified gene predictors were carried out. Based on survival information from a total of 479 LUAD samples, the risk score was stratified into high and low groups.

The Kaplan–Meier survival estimation with the log-rank test, a typical non-parametric method (Murray and Tsiatis, 1996; Royston et al., 2008), was adopted to predict the survival probability on all corresponding LUAD samples from TCGA.

**Figure 6:**
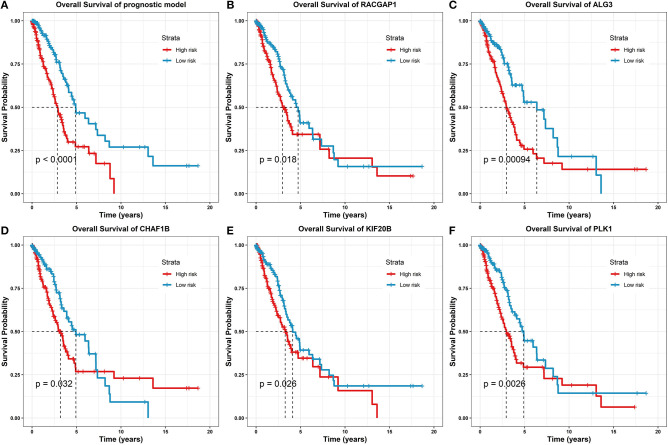
Survival analysis of the prognostic model and gene predictors. **(A)** On the prognostic model, where the high-risk group marked as red line had a significantly lower survival rate than the low-risk group marked as blue (log-rank test *p*-value < 0.0001). **(B)** On the predictor, RACGAP1 (log-rank test *p*-value = 0.018). **(C)** On the predictor, ALG3 (log-rank test *p*-value = 0.00094). **(D)** On the predictor, CHAF1B (log-rank test *p*-value = 0.032). **(E)** On the predictor, KIF20B (log-rank test *p*-value = 0.026). **(F)** On the predictor, PLK1 (log-rank test *p*-value = 0.0026).

Furthermore, we validated the eight gene predictors in the prognostic model, and we examined whether these genes were capable of independently predicting prognostic survival. The analysis results are illustrated in Figure 6.

From the survival analysis results, the prognostic model was statistically significantly correlated with the clinical outcomes in LUAD (log-rank test *p*-value < 0.0001); together, 5/8 of the prognostic predictors have statistically significant clinical importance (log-rank test *p*-values ranging from 0.032 to 0.00094). For the other predictors, due to the *p*-value > 0.05, the survival analyses are given in Supplementary Material.

### Performance Comparison With Classic Least Absolute Shrinkage and Selection Operator Methods

To validate the method efficiency, the proposed FSOR method was compared in feature selection and prediction capability with classic LASSO regression, in terms of the ROC curve and corresponding AUC measure, respectively.

**Figure 7:**
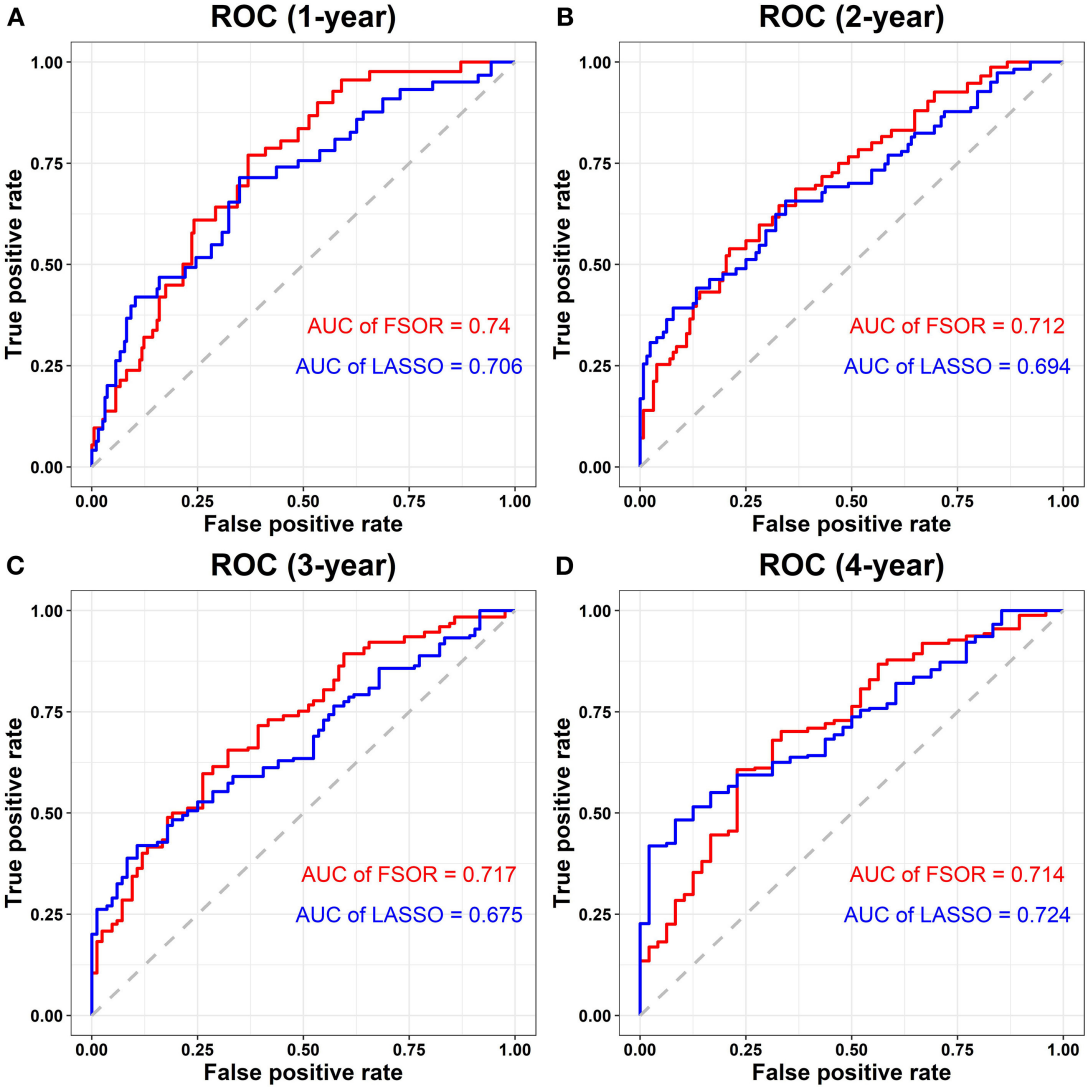
Performance comparison on risk score survival analysis with the feature selection with orthogonal regression (FSOR) and classic least absolute shrinkage and selection operator (LASSO)–Cox regression models. **(A)** Comparative receiver operating characteristic (ROC) for the 1-year term. **(B)** Comparative ROC for the 2-years term. **(C)** Comparative ROC for the 3-years term. **(D)** Comparative ROC for the 4-years term.

Figure 7 depicts the performance comparison results based on the measure AUC by FSOR-Cox and LASSO-Cox methods.

From the above comparisons on the averaged 1- to 4-years AUC measure, we found that the proposed FSOR outperforms the classic LASSO methods, namely, 0.7208 vs. 0.6998. Specifically, for the 1- to 3-years AUC measure, it has significant advantages over the classic ones in the enhanced prediction performance, indicating that the former has a certain potential in the early-stage prognosis application.

## Discussion and Conclusion

Till now, carcinoma diagnosis and prognosis are facing substantial difficulties in acquiring effective clinical model and enhanced prediction performance. To address the key problem in feature screening and to improve the prognostic model performance, we proposed a novel FSOR method in this study.

The method is primarily to solve a constrained minimization problem by an ALM approach and, thus, to optimize feature selection and LASSO regression from gene profiling data.

Together with integrative analyses on the biological function (PPI) and physical network property, it revealed that the identified candidate predictors had a significant concordance in their biological function and network clique centrality property, partially proving the reliability of the candidate predictors.

Furthermore, clinical outcome prediction and robustness evaluation were conducted on the constructed prognostic model and individual gene predictor, respectively. The results on multi-omics data of LUAD demonstrated the proposed FSOR method outperformed more accurately by 0.7208 with fewer predictors than classic LASSO regression models (the averaged 1- to 4-years predictive AUC measure, 0.6998, on TCGA clinical data). Particularly, its averaged 1- to 3-years AUC reaches 0.723, vs. classic 0.6917.

From the ROC curve distribution, it is obvious that the prediction performance of the proposed FSOR prognostic model is significantly higher than that of classic LASSO approaches; from the clinical outcome perspective, the results validated the feasibility of the FSOR method to screen candidate predictors with better prognostic performance.

For clinical research and application, the proposed FSOR is easily utilized and adopted due to its consolidated methodology and open-sourced scripts. We thoroughly tested and validated on the real experiment and cohort data sources from GEO and TCGA. Furthermore, to a broader perspective, the proposed method has the potential scalability to other cancer and disease types.

In conclusion, the proposed FSOR method can deliver better prediction performance for the early-stage prognosis and has the potential to improve therapy strategy, but with few predictor consideration and computation burden. The future work should focus on integrating multi-omics and multi-scale profiling information (Tang et al., 2017), together with proposing novel analytical approaches (Liu et al., 2020; Qi et al., 2020), thus to optimize therapy targets and boost precision medicine.

## Data Availability Statement

The gene profiling data used in this study are available at GEO with the accession IDs GSE32036, GSE32867, GSE33532, and GSE75037; other gene profiling and clinical information are available at TCGA GDC portal. The self-composed scripts utilized in the study are available at GitHub (https://github.com/gladex/PM-FSOR).

## Author Contributions

BT and YW conceived the study, performed the analyses, and wrote the manuscript. YC, ML, and YT suggested and modified the analyses. All authors read and approved the final manuscript.

## Conflict of Interest

The authors declare that the research was conducted in the absence of any commercial or financial relationships that could be construed as a potential conflict of interest.
