# Supplementary material for: A Novel Early-Stage Lung Adenocarcinoma Prognostic Model Based on Feature Selection With Orthogonal Regression
Source: Front Cell Dev Biol. 2021 Jan 8;8:620746. doi: 10.3389/fcell.2020.620746 (PMC7874010; doi:10.3389/fcell.2020.620746)
Supplement: Supplementary file 1 [file Data_Sheet_1.PDF]

Supplementary File

Binhua Tang, Yuqi Wang
Contact: bh.tang@outlook.com

1. The pseudocode of the GPI method.

Nie et al. [1] proposed a novel generalized power iteration (GPI) method for solving this quadratic problem on the Stiefel manifold,

GPI method

1. **Input:** the symmetric matrix $C \in R^{d\times d}$ and the matrix $D \in R^{d\times k}$
2. **Initialize:** a random $W \in R^{d\times k}$ such that $W^T W = I_k$ and $\tilde{C} = \alpha I_d - C$ where α is the dominant eigenvalue of C.
3. **Repeat**
4. Update $N \leftarrow 2\tilde{C}W + 2D$.
5. Calculate $USV^T = N$ via the compact SVD method of N.
6. Update $W \leftarrow UV^T$
7. **Iteratively perform** the steps 4-6 until converged
8. **Output:** the matrix $W \in R^{d\times k}$

2. The pseudocode of AML

The constrained minimization problem can be solved by an augmented Lagrangian multiplier (ALM) method [2], as below,

ALM method

1. **Input:** matrix $H \in R^{d\times d}$, vector $r \in R^{d\times 1}$.
2. **Initialize:** $\rho > 1, \phi_i = \frac{1}{d}(1 \le i \le d), v = \phi, \lambda_2 = 0, \mu > 0, \lambda_1 = (0,0,...0)^T \in R^{d\times 1}$.
3. **Repeat**
4. Update J by $J = 2H + \mu I_d + \mu \mathbf{1}_d \mathbf{1}_d^T$
5. Update g by $g = \mu v + \mu \mathbf{1}_d - \lambda_2 \mathbf{1}_d - \lambda_1 + r$
6. Update $\hat{\phi}$ by $\hat{\phi} = J^{-1} g$
7. Update v by $\hat{v} = f(\hat{\phi}, \mu, \lambda_1)$
8. Update λ_1 by $\lambda_1 = \lambda_1 + \mu(\hat{\phi} - v)$
9. Update λ_2 by $\lambda_2 = \lambda_2 + \mu(\phi^T \mathbf{1}_d - 1)$
10. Update μ by $\mu = \rho\mu$
11. **Iteratively perform** the steps 4-10 until converged
12. **Output:** the weights vector ϕ

3. The Protein-protein interaction (PPI) network of top 50 genes selected from the FSOR

Based on the FSOR analysis, the top 50 genes were selected from its output weighted matrix. To ensure the underlying functional association among the selected genes, protein-protein interactions were further predicted with STRING [3], depicted as below,

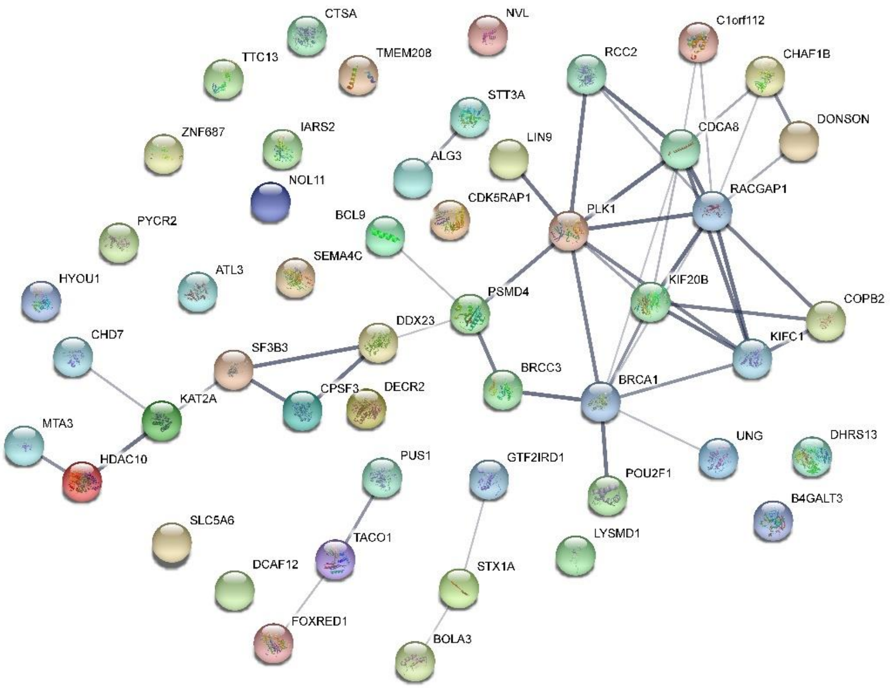

4. Summary table of 32 candidate genes, with the FSOR-derived weight information.

From the derived PPI network, we chose the gene nodes with at least one edge connected within the network, thus totally 32 genes were identified as candidate genes, detailed as below,

Gene symbol	Entrez ID	Weight coefficient
CPSF3	51692	22.18467
UNG	7374	20.9224
CDCA8	55143	20.20573
RCC2	55920	19.39025
PLK1	5347	18.67585
DDX23	9416	18.33255
TACO1	51204	17.97514
COPB2	9276	16.67461
STX1A	6804	15.72301
POU2F1	5451	15.52422
SF3B3	23450	15.42259
STT3A	3703	15.24171
KAT2A	2648	15.1746
PSMD4	5710	14.9895
MTA3	57504	14.88998
KIF20B	9585	14.60656
KIFC1	3833	14.52312
C1orf112	55732	13.60323

RACGAP1	29127	13.21505
BRCC3	79184	12.93091
DONSON	29980	12.78419
GTF2IRD1	9569	12.38783
ALG3	10195	12.09634
CHAF1B	8208	12.06833
LIN9	286826	11.93583
BCL9	607	11.37583
PUS1	80324	11.37543
HDAC10	83933	11.09244
CHD7	55636	10.98454
FOXRED1	55572	10.87981
BRCA1	672	10.87947
BOLA3	388962	10.75333

5. Survival analysis on gene predictors in constructing the prognostic model

From the survival analysis results, the prognostic model was statistically significantly correlated with the clinical outcomes in LUAD (log-rank test p-value < 0.0001), together the 5/8 of the prognostic predictors have statistically significant clinical importance, respectively (log-rank test p-values ranging from 0.032 to 0.00094). For the other 3/8 predictors, due to the p-value > 0.05, the survival analyses are depicted as below,

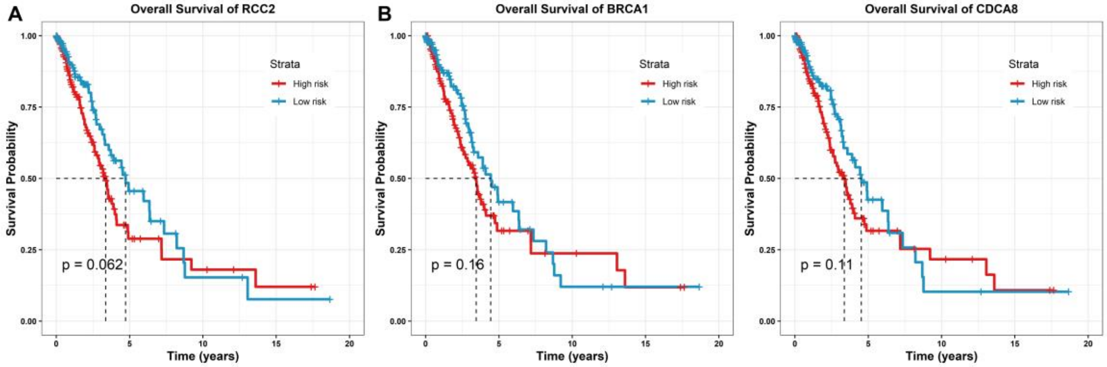

References

1. Nie F, Zhang R, Li X: **A generalized power iteration method for solving quadratic problem on the Stiefel manifold.** *Science China Information Sciences* 2017, **60:**112101.
2. Hu S, Wang J, Huang Z-H: **An inexact augmented Lagrangian multiplier method for solving quadratic complementary problems: An adapted algorithmic framework combining specific resolution techniques.** *Journal of Computational and Applied Mathematics* 2019, **361:**64-78.
3. Szklarczyk D, Gable AL, Lyon D, Junge A, Wyder S, Huerta-Cepas J, Simonovic M, Doncheva NT, Morris JH, Bork P, et al: **STRING v11: protein–protein association networks with increased coverage, supporting functional discovery in genome-wide experimental datasets.** *Nucleic Acids Research* 2018, **47:**D607-D613.
